# Impact of Work Value Perception on Workers’ Physical and Mental Health: Evidence from China

**DOI:** 10.3390/healthcare9081059

**Published:** 2021-08-18

**Authors:** Fan Yang, Yao Jiang, Xiaohong Pu

**Affiliations:** 1 Department of Labor and Social Security, School of Public Administration, Sichuan University, Chengdu 610065, China; yangfan1987@scu.edu.cn; 2 Department of Sociology, Zhou Enlai School of Government, Nankai University, Tianjin 300350, China; s20175706@stu.sicau.edu.cn

**Keywords:** ERG theory, life satisfaction, labor welfare, labor economics, health policy

## Abstract

Research on the effect of work value perception on workers’ health, especially in emerging economies, is scarce. This study, therefore, explored how work value perception affects the physical and mental health of workers in China. We also examined the mediating role of life satisfaction in the relationship between work value perception and health. Taking a random sample of 16,890 individuals in China, we used ordered probit regression and instrumental variable ordered probit regression to test the links between work value perception and workers’ health based on existence, relatedness, and growth (ERG) theory. The results showed that work value perception significantly affected both the physical and mental health of workers; the results remained robust after solving the endogeneity problem. The subsample regression results showed that work value perception significantly affected the physical and mental health of female, male, married, unmarried, religious, and nonreligious workers. Furthermore, life satisfaction mediated the effect of work value perception on workers’ health. These results shed light on the relationship between work value perception and health and thus have implications for improving workers’ physical and mental health. This study can provide a reference for both governmental and corporate policymakers in emerging economies.

## 1. Introduction

The ancient Greek philosopher Socrates suggested that “the unexamined life is not worth living” [1]. Similarly, it is important for workers to recognize the value of their work since work value perception has been found to strongly affect workers’ outcomes, both at work and beyond [2,3,4,5,6,7]. Work value perception generally refers to workers’ perception of the value category, such as economic value, emotional value, social value, respect value, ability value, and interest value, and these above values in regard to the work that they are engaged in [8,9,10]. Workers’ health has recently become an important topic in the field of labor economics [11,12,13,14,15]. Workers’ health is not only related to labor productivity and economic development [16,17,18], but also is a crucial aspect of labor welfare [19,20,21,22,23]. There is, however, a lack of consensus on the factors that influence workers’ health. While certain classic material-level factors, such as work protection, have been extensively investigated [24,25,26,27,28], various hidden factors, such as spiritual-level factors, have not been deeply explored. It is known that workers, especially in low-skilled positions, do not always recognize the value of their work [29]. Some workers, for example, may view their work as simply a way to make a living and nothing more [30]. Such workers can be figuratively compared to machines on an assembly line [31,32]. Therefore, the question of whether work value perception affects workers’ physical and mental health is worth investigating.

This study aimed to determine the effects of work value perception on the physical and mental health of workers in China, which is a major emerging economy. We also tested the mediating role of life satisfaction. In this way, we connected work value perception with individual needs and established a theoretical framework for investigating the relationship between work value perception and health. By examining this issue empirically using a nationally representative sample of 16,890 workers, this study opens up new directions for research in a fast-growing field that has important implications for policymakers.

This study extends existing knowledge in three ways. First, by investigating the effect of work value perception on workers’ physical and mental health, this study enriches research on the factors that influence workers’ health. Second, we address the endogeneity problem between work value perception and health and identify the net effect of work value perception on workers’ health. Third, by using big data, we offer wide-ranging policy implications for improving the physical and mental health of Chinese workers.

## 2. Theoretical Framework and Hypothesis Development

Having a sense of value is an important part of people’s lives, and the pursuit of value is a driving force in both work and life. The purpose of pursuing value in work and life is to meet individuals’ needs. Based on Maslow’s hierarchy of needs, Alderfer developed the theory of “existence, relatedness, and growth” (ERG), which proposes that people have three basic types of needs: existence needs, relatedness needs, and growth needs [33].

Existence needs are lower-order needs related to physiological needs and the need for safety. Relatedness needs concern interpersonal relationships and the desire for social status. Growth needs, meanwhile, correspond to Maslow’s need for self-actualization [33,34]. Similar to Maslow’s hierarchy of needs, the three basic types of needs in the ERG theory also have a hierarchy. Generally, existence needs are met first, relatedness needs are followed, and growth needs are met finally. An empirical investigation by Alderfer suggested that ERG could produce more accurate descriptions of the outcomes of employees’ needs and desires compared to Maslow’s theory [33,34].

The perception of value that a job brings to workers corresponds to their needs [35]. If workers feel that their job meets their needs, they may derive a sense of satisfaction, which may in turn yield benefits, such as good mental and physical health [36]. If, however, workers do not see value in their work, there may be adverse consequences, such as negative emotions and a loss of meaning in work and life.

### 2.1. Work Value Perception Related to Existence Needs and Health

According to ERG theory, a job should first meet workers’ existence needs [33]. Work value perception is associated with existence needs and includes economic and emotional value perception. Economic value perception responds to physiological needs, and emotional value perception responds to safety needs. The economic value perception of a job refers to the sense of the realization of the economic benefits brought by work. The economic value of work provides the material basis for workers to maintain their physical and mental health [35,37]. When workers face economic pressure, anxiety and other negative emotions can arise and, in turn, adversely affect physical and mental health [38]. Furthermore, when workers unexpectedly become ill or mentally distressed, the economic benefits of work can help them to seek medical treatment or counseling to restore them physically or mentally and thereby help to prevent mental or physical deterioration [39]. Therefore, the economic value of a job and the degree of workers’ perception of this economic value may affect their physical and mental health.

In terms of emotional value perception, the positive emotions brought about by a job are key to workers’ health [40]; this is similar to the effects of positive emotions on people’s health in daily life. In general, positive emotions are considered beneficial for health [41]. If people experience negative emotions because of work-related matters, it is likely to have adverse effects on their physical and mental health, leading to, for example, obesity [42] or anxiety [43,44] by negatively affecting their health-related lifestyles, such as diet and drinking [45,46]. In short, the emotional value perception of a job may have an effect on workers’ physical and mental health.

### 2.2. Work Value Perception Related to Relatedness Needs and Health

Relatedness needs in ERG theory are concerned with interpersonal relationships and the desire for status—namely social needs and the need for respect [33,34]. Therefore, based on relatedness needs, a job should bring social value and respect value to workers. Regarding the social value perception of work, some studies have confirmed the benefits of good socializing at work [47,48]. First, good interaction at work makes it easier for workers to integrate into the work environment and can support their expression, adaptation, acceptance, and coordination [49]. Second, good interaction at work helps to establish good relations with colleagues and employers and can bring promotion opportunities [50]. Third, good interaction at work helps to create a good work environment, improve work efficiency, and produce high work performance [51]. Lastly, good interaction at work supports the formation of friendships with partners or customers and the building of wide interpersonal networks [52]. These benefits can support workers’ physical and mental health, either by affecting the material conditions for maintaining physical health or by affecting emotions related to maintaining mental health.

Regarding respect value perception at work, workers take delight in gaining recognition and respect from colleagues [53]. This is a type of nonmaterial psychological or spiritual motivation that can help workers to build work confidence, avoid burnout, and be confident when facing challenges [54]. Thus, respect value perception can improve workers’ mental health by supporting their internal, nonmaterial needs. If, however, workers are not treated with respect in the workplace, it may adversely affect their mental health by harming their sense of dignity, achievement, and self-identification [55].

### 2.3. Work Value Perception Related to Growth Needs and Health

Growth needs are related to self-actualization in terms of ability and interest [33,34]. They occupy the highest position in the hierarchy in the ERG theory. In this regard, a job should respond to workers’ needs for self-actualization and should give them work value perception related to growth, including ability value perception and interest value perception. Work value perception related to growth needs can also produce many benefits for workers. Regarding ability value perception, giving full play to workers’ abilities can generate good work performance, cultivate a sense of self-worth [56,57,58], and foster a positive mood, all of which are conducive to workers’ physical and mental health.

Regarding interest value perception, modern education aims to develop people’s career choices based on their interests [59,60,61]. Studies have found that the congruence between interests and occupation is a crucial factor affecting job satisfaction [62,63,64,65]. Further, job satisfaction can have significant effects on health, especially mental health (e.g., burnout, self-esteem, depression, anxiety) [66,67,68,69]. Therefore, we speculate that the perception of interest value may affect workers’ physical and mental health.

Based on the above analysis of work value perception related to ERG, we propose that work value perception is closely related to workers’ physical and mental health. Thus,

**Hypothesis 1** (**H1**)**.** *Work value perception will affect workers’ physical health.*

**Hypothesis 2** (**H2**)**.** *Work value perception will affect workers’ mental health.*

### 2.4. Mediating Role of Life Satisfaction

Life satisfaction refers to an individual’s overall subjective evaluation of his or her life [70,71]. Work plays a central role in daily life because it greatly determines the economic basis of a person’s life [72,73,74]. Therefore, an individual’s value perception of their work—including the perception of economic, emotional, social, respect, ability, and interest value—is likely to influence life satisfaction by affecting career adaptability [75], job burnout [76], and work satisfaction [77,78]. Furthermore, life satisfaction can affect people’s moods and health behaviors, including smoking, alcohol consumption, exercise, and diet [79,80,81]. Thus,

**Hypothesis 3** (**H3**)**.** *Life satisfaction will mediate the effect of work value perception on workers’ physical health.*

**Hypothesis 4** (**H4**)**.** *Life satisfaction will mediate the effects of work value perception on workers’ mental health.*

**Figure 1:**
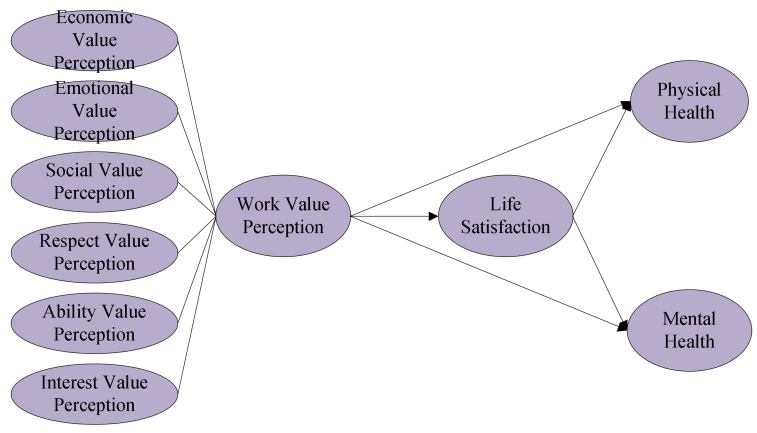
Research model.

Given H1–H4, Figure 1 presents our research model, describing the anticipated relationships between work value perception and workers’ physical and mental health.

## 3. Method

### 3.1. Sample

We used the China Labor-Force Dynamics Survey 2016 (CLDS2016) dataset to test the proposed model. In the CLDS2016, workers are defined as laborers who are employed by other individuals or organizations or self-employed. The survey was conducted from July to September 2016 by the Center for Social Science Survey at Sun Yat-sen University. The respondents answered the survey questionnaire anonymously and voluntarily. The survey was nationally representative, and it collected data from 29 provincial administrative units (excluding Hong Kong, Macao, Taiwan, Tibet, and Hainan). Respondents included rural workers (whose residence (Hukou in Chinese) was registered in rural areas and worked also in rural areas), urban workers (whose Hukou was registered in urban areas and worked also in urban areas), and rural–urban migrant workers (whose Hukou was registered in rural areas but worked in urban areas). We cleaned the data and deleted invalid datasets with too many missing answers. Finally, 16,890 samples were included in the analysis. Among them, 34.66% of participants were employed or self-employed in a primary industry (including agriculture, forestry, husbandry, and fishery), 15.51% in a secondary industry (including the industry sector and the construction industry), and 49.83% in a tertiary industry (including transport, storage and post, wholesale and retail trades, hotels and catering services, and emerging industries such as financial intermediation, real estate, and other services).

### 3.2. Measures

#### 3.2.1. Explained Variables

The explained variables of this paper are the physical and mental health of workers. For physical health, referring to previous studies [82,83,84], we used self-rated health to measure respondents’ physical health status. Respondents were asked, “How do you evaluate your current physical health status?” The answer was measured on a five-point Likert scale. “Very unhealthy” was coded as 1, “somewhat unhealthy” as 2, “normal” as 3, “somewhat healthy” as 4, and “very healthy” as 5.

For mental health, we used the Center for Epidemiological Studies Depression Scale (CES-D), developed by Radloff [85], to measure respondents’ mental health outcomes. The CES-D is one of the most widely used tools for assessing depression and mental health [86,87]. A study focusing on Chinese mental health also used the scale and found it effective [88]. It has 20 items, each scored on a four-point Likert scale, except for items 4, 8, 12, and 16 (1 = rarely or none of the time (<1 day), 2 = some or a little of the time (1–2 days), 3 = occasionally or a moderate amount of the time (3–4 days), 4 = most or all of the time (5–7 days)). For questions 4, 8, 12, and 16, the score is the same but reversed: “Most or all of the time (5–7 days)” is scored one point, and “Rarely or none of the time (<1 day)” is scored four points. The total score ranges from 20 to 80. Generally speaking, the higher the CES-D score, the deeper the depression, and the worse the mental health status.

We tested the validity of the CES-D (Appendix A). Polychoric correlation coefficient results show that the minimum correlation coefficient among the items of CES-D is 0.466. It indicates that the 20 items have good validity to measure the mental health of Chinese workers.

#### 3.2.2. Explanatory Variable

The explanatory variable of this paper is the work value perception of workers. According to the previous theoretical framework, we used six dimensions (Appendix B) to measure workers’ work value perception. Specifically, we used economic value and emotional value perception for existence needs, social value and respect value perception for relatedness needs, and ability value and interest value perception for growth needs. Each dimension was measured on a five-point Likert scale. An answer of “strongly disagree” was coded as 1, “disagree” as 2, “normal” as 3, “agree” as 4, and “strongly agree” as 5. The level of workers’ work value perception was obtained by aggregating the answers to these six questions. In the empirical part, we also analyzed the impact of each dimension of work value perception on workers’ physical and mental health.

#### 3.2.3. Mediating Variable

According to the theoretical framework, the mediating variable of this paper is life satisfaction. We measured life satisfaction with the question, “Are you satisfied with your current life?” [76,89]. It was measured on a five-point Likert scale (1 = “very dissatisfied,” 2 = “somewhat dissatisfied,” 3 = “normal,” 4 = “somewhat satisfied,” 5 = “very satisfied”). Previous studies have confirmed this to be a reliable measure of life satisfaction [74,90,91].

#### 3.2.4. Control Variables

To draw accurate conclusions, we controlled general factors that might have affected workers’ health, including demographic characteristics such as gender, age, education, marital status, and religion [92,93,94,95,96,97,98]; socioeconomic status—income [99]; and lifestyle habits such as smoking, drinking, and exercise [100,101]. We also controlled the fixed effect of region according to the provinces where the respondents lived.

### 3.3. Data Analysis

The explained variables were the physical and mental health of workers. Workers’ work value perception was the explanatory variable. The physical health of workers represented discrete and ordered data, and ordered probit regression could therefore be used to estimate the influence of work value perception on physical health. This study’s ordered probit model can be written as follows:

(1) $${Physical\;health}^{*}=\beta x+\gamma Z+\varepsilon$$

where ${Physical\;health}^{*}$ is the explained variable, representing workers’ physical health; x is workers’ work value perception; Z represents a set of control variables that might affect workers’ physical health; β and γ are the coefficient vectors; and ε is the error term.

In the model, ${Physical\;health}^{*}$ cannot be observed directly; however, Physical health can be observed. We assumed the selection rule of Physical health as follows:

(2) $$Physical\;health=\left\{\begin{array}{l} {=1\;If\;Physical\;health}^{*}\leq 1\\ {=2\;1<Physical\;health}^{*}\leq \mu_{1}\\ {=3\;\mu }_{1}{<Physical\;health}^{*}\leq \mu_{2}\\ \ldots \;\ldots \\ {=J\;Physical\;health}^{*}\geq \mu_{J-2} \end{array}\right.,$$

where $\mu_{1}{<\mu }_{2}<\ldots \leq \mu_{J-2}$ are unknown parameters estimated together with β and γ.

Meanwhile, workers’ mental health is a continuous variable. Therefore, we used ordinary least-squares (OLS) regression to estimate the influence of work value perception on workers’ mental health. The OLS model used in this study can be written as follows:

(3) $${Mental\;health=\alpha }_{0}+\delta x\;+\;\vartheta Z\;+\;\epsilon$$

where Mental health= is the explained variable, meaning workers’ mental health; x and Z have the same meanings as in Equation (1); $\alpha_{0}$ represents the intercept item; δ and ϑ are the coefficients; and ϵ is the error term.

An endogeneity problem between work value perception and workers’ physical or mental health may have existed in the ordered probit and OLS models, leading to estimation error. The reasons are as follows.

First, workers’ work value perception was the result of self-selection rather than random selection. This means that work value perception may have been affected by other unobserved factors that also affected workers’ physical or mental health. Thus, selection bias was present.

Second, work value perception and workers’ physical or mental health may have had relationships of mutual causality. As we proposed, work value perception might have affected workers’ physical and mental health. However, workers with good physical and mental health may have had more positive attitudes toward the value of the work that they were engaged in.

Third, since physical and mental health in this study were both self-rated measurements, they may have contained bias, which also might have created an endogeneity problem.

Fourth, some unobserved factors affecting workers’ physical and mental health also might have caused endogeneity. Although general factors were controlled in the model, there still might have been missing variables.

Since the traditional ordered probit and OLS models cannot solve the abovementioned endogeneity problems, we used an instrumental variable (IV) ordered probit model and an IV two-stage least-squares (IV−2 SLS) model to address the endogeneity problem.

Based on peer effect, we used the mean value of workers’ work value perception in the same community except for worker i as the IV. Theoretically, the mean value of workers’ work value perception in the same community except for worker i is a qualified IV, and it meets the two requirements of IV: relevance and exclusion. The work value perception of workers in the same community might strongly influence other workers, resulting in similar work value perception among them. Thus, the IV used in this study was closely correlated with the explanatory variable (i.e., workers’ work value perception). In addition, based on peer effect, the community members of the same community are similar in aspects of economic status and values, while community members of different communities in those aspects are heterogeneous. Similarly, the work value perception in different communities may be heterogeneous. Further, the mean value of workers’ work value perception in the same community except for worker i does not have a direct association with workers’ physical and mental health. Therefore, the IV was appropriate.

Moreover, we used bootstrap estimation to elicit the influence mechanism of work value perception’s effect on physical and mental health and test the mediating effect of life satisfaction.

## 4. Results

### 4.1. Descriptive Analysis

Table 1 shows the results of the descriptive analysis. With regard to the explained variables, physical health and mental health, the average value for physical health was 3.589 (SD = 0.994) on a scale of 1–5. Meanwhile, the mean value for mental health was 27.121 (SD = 8.899) on a scale of 20–80. The explanatory variable (work value perception) ranged from 6 to 30, with a mean value of 21.859 (SD = 4.126).

With regard to the control variables, 50.2% of the respondents were male, indicating that gender was distributed fairly evenly. Age ranged from 16 to 96 years, with a mean value of 46.858 (SD = 12.898). Interestingly, the fact that the highest age in the sample was 96 suggests that, in China, engagement in certain types of work might not have maximum age restrictions. The average amount of education was 8.658 (SD = 4.200) years, with a minimum of 0 years (illiteracy) and a maximum of 23 years (doctoral degree). A total of 87.4% were married, and 12.7% held religious beliefs. The logarithm of annual income had a mean value of 9.910 (SD = 1.141) in 2015. In terms of lifestyle, 30.80% smoked, 22.22% drank alcohol, and 31.40% exercised regularly.

### 4.2. Benchmark Regression

We used ordered probit and OLS models as the benchmark regressions to estimate the effects of work value perception on workers’ physical and mental health, respectively. Table 2 shows the results.

For physical health, column (1) in Table 2 shows the influence of work value perception on workers’ physical health, estimated without control variables. The results in column (1) show that work value perception was significantly associated with workers’ physical health at the 1% significance level. The results in column (2) show estimations similar to those in column (1) after controlling for regional fixed effects. In column (3), work value perception still has a statistically significant correlation with workers’ physical health at the 1% level when regional fixed effects and respondents’ characteristics are controlled. These results indicate that the more strongly workers recognized the value of their work, the better their physical health. Thus, H1 is supported.

For mental health, column (4) in Table 2 reports the effect of work value perception on workers’ mental health estimated without control variables. The results show that workers’ work value perception significantly affected mental health. Similarly, the results in columns (5) and (6) still align with those in column (4) after adding the control variables. The higher value of work value perception indicates the greater work value workers perceived from the work that they engaged in; meanwhile, the larger score of CES-D indicates greater depression and worse mental health. Thus, the results in Table 2 show that the work value perception and mental health change in a reverse trend, which indicates that the higher the work value perception, the better the mental health outcomes. Therefore, H2 is supported.

Regarding the control variable results in columns (3) and (6) in Table 2, the effects of the control variables were mostly consistent with the findings of previous studies. Specifically, physical and mental health both declined with age, while marriage had a positive influence on physical and mental health. Moreover, religious belief was significantly associated with mental health, and lifestyle (drinking, exercise) significantly affected physical health. Further, life satisfaction positively influenced both physical and mental health.

In summary, the results of the ordered probit and OLS benchmark regressions aligned with the theoretical analysis; that is, high work value perception could significantly improve workers’ physical and mental health outcomes. The endogeneity problem in the benchmark regressions was not addressed, however, and the results in Table 2 might have estimation errors. Therefore, we added IV to the ordered probit and OLS models to further explore the relationships between work value perception and workers’ physical and mental health.

### 4.3. Solving the Endogeneity Problem

To address the endogeneity problem, we used IV-ordered probit and IV−2 SLS models for estimation. The results in Table 3 show that after controlling for endogeneity, work value perception was still significantly correlated with workers’ physical and mental health, which is consistent with the benchmark regression results in Table 2.

We conducted IV as a diagnostic test. The F-values of the IV-ordered probit and IV−2 SLS models were both far greater than 10. This means that the IV used in this study was strong. Moreover, in the first-stage regression, we found a high R^2^, and in the second stage, when IV was included in the IV-ordered probit and IV−2 SLS models, the coefficients of IV were both insignificant. These results indicate that the proposed IV satisfied the conditions of relevance and excludability, and the results were thus reliable.

After adding the IV into the models, the effects of the control variables were also consistent with the results in Table 2. The variables of age, education, marital status, income, and life satisfaction were significantly associated with individual physical and mental health.

### 4.4. Regressions of Different Dimensions

In order to observe the impact of the six dimensions of work value perception (economic, emotional, social, respect, ability, and interest value perception) on workers’ physical and mental health, we conducted regressions of different dimensions. As can be observed from the results estimated by the IV-ordered probit model in Table 4, the six work value perception dimensions all impacted workers’ physical health positively and significantly. This indicates that the higher the perception of the six dimensions of work value, the better the physical health of workers. Further, we found that the coefficients of economic, emotional, social, respect, ability, and interest value perception on the physical health of workers decreased progressively.

The impacts of the six work value perception dimensions on worker’s mental health are presented in Table 5, as estimated by IV−2 SLS. The results show that the six work value perception dimensions correlate to workers’ mental health negatively and significantly, which means that workers with higher perception of the six dimensions of work value tended to have a lower CES-D score and were less likely to suffer from depression. Similar to the results of physical health, the impacts of economic, emotional, social, respect, ability, and interest value perception on the mental health of workers ranged from strong to weak gradually.

### 4.5. Robustness Check by Subgroup Regressions

To check the robustness of the regression results, we further explored the effects of work value perception on workers’ physical and mental health according to different gender, marital status, and religious belief groups. The full samples were grouped into six subgroups: female/male, married/unmarried, and religious/nonreligious. We also estimated the subsample regression models using IV-ordered probit and IV−2 SLS models. Table 6 and Table 7 report the results.

The results in Table 6 show that work value perception positively affected the physical health of all workers belonging to different gender, marital status, and religion subgroups. This means that the full sample results were highly robust—that is, the greater the work value perception, the better the physical health results.

The results in Table 7 show that work value perception also had positive effects on workers’ mental health among different gender, marital status, and religion subgroups. In short, the greater the work value perception, the lower the likelihood of depression. Therefore, our results were robust.

### 4.6. Mechanism Analysis

We used bootstrap estimation to test the significance of the mediating effect of life satisfaction. Bootstrap samples were drawn 500 times to test the mediating effects. Table 8 and Table 9 show the direct and indirect effects of life satisfaction on the total sample’s and the subgroups’ physical and mental health, respectively. The 95% confidence intervals of all subgroups for the effects of work value perception on physical and mental health did not overlap. Because of space limitations, we do not report the results of the 95% confidence intervals in Table 8 and Table 9.

For physical health, in Table 8, the direct and indirect effects of life satisfaction on the total sample’s and the subgroups’ physical health are significant at the 1% level. This means that life satisfaction mediated the effect of work value perception on the total sample’s and the subgroups’ physical health. Thus, H3 is supported.

For mental health, in Table 9, the direct and indirect effects of life satisfaction on the total sample’s and the subgroups’ mental health were significant at the 1% level. This means that strong work value perception significantly reduced the likelihood of depression and enhanced mental health via life satisfaction. Thus, H4 is supported.

## 5. Discussion

### 5.1. Theoretical Implications

Based on ERG theory, this study investigated the impact of work value perception on workers’ health. The results suggest that work value perception is an influential antecedent of self-rated physical health and mental health among Chinese workers, which is in line with the expectations in the theoretical framework of this paper. This study expands the theory of labor health and welfare in labor economics by validating a model investigating the effects of work value perception on the self-rated physical and mental health of Chinese workers.

First, we add another layer of understanding to the determinants of workers’ health in the Chinese context. Many studies have found that labor protection at the material level [27] and work incentive at the spiritual level [102] are important factors affecting physical and mental health. This study indicated that work value perception also plays an important role in workers’ life satisfaction, which in turn has significant beneficial effects on physical and mental health.

Second, in the further regressions of different dimensions, this study explores the different impacts of the six dimensions of work value perception on the health of workers. The impacts of economic, emotional, social, respect, ability, and interest value perception on the physical and mental health of Chinese workers decreased progressively. As such, in the Chinese context, the economic value that workers perceived from the work that they engaged in occupied the highest position, while interest value occupied the lowest position in the six dimensions of work value perception derived from the ERG theory. The hierarchy of the six dimensions of work value perception is confirmed in this study, which is consistent with the ERG theory.

Third, examining workers’ physical and mental health in terms of psychology and value perception extends the literature, especially in the context of rapid artificial intelligence (AI) development, which warrants research attention. With machines increasingly replacing workers, the intrinsic value of certain types of workers has been undermined, producing negative results, including a deterioration in health. The question of how to build a good society in the AI era has thus become a topic of research interest [103]. Thus, academia should pay attention to the work value perception of workers. This study contributes to this rising trend in labor economics research regarding the health and welfare of workers as well as the related influencing factors.

### 5.2. Practical Implications

This study’s findings have implications for both enterprise managers and government policymakers. For firms, we have shown that work value perception should be considered when formulating employee health and welfare policies. Specifically, when carrying out vocational training, work value perception should be emphasized so that employees can fully realize the economic, emotional, social, respect, ability, and interest value brought by their work. Moreover, when arranging positions, employees’ wishes with regard to job choice should be respected within the scope of their abilities. They should be allowed to work in positions that are consistent with their self-recognition of work value.

For government decisionmakers, this study shows that work value perception can strongly affect workers’ life satisfaction and improve their physical and mental health. Therefore, when considering ways to improve public health, labor productivity, and social prosperity, government decisionmakers should consider adopting policies to increase work value perception among workers. Specifically, attention should be paid to educating the public in this regard before they enter the workplace. The government could also introduce ways to supervise enterprises’ efforts to improve employees’ work value perception.

Specifically, the work value perception of workers may be improved in terms of the six dimensions, including the perception of economic, emotional, social, respect, ability, and interest value. For economic value perception, gradually increasing workers’ income is the key driver to improving their perception of the economic value of their work. For emotional value perception, it is necessary to conduct a person–job fit test for workers before employment. The employer can consequently arrange for workers with different personalities to engage in work that matches their personality in order to ensure that they maintain positive and stable emotions. For social value perception, appropriately providing visits and communication opportunities with peers for workers at work may contribute to the improvement of their social value perception. For respect value perception, superiors should treat subordinates with respect, and peers should respect each other in the workplace, which not only requires conscious effort from all involved but also requires sound rules to be established for effective supervision. For ability value perception, timely recognition of workers’ good work performance may help to improve their confidence in their work ability. For interest value perception, on the one hand, individuals should constantly develop their interests in their work. On the other hand, it is vital to ensure that the job position and work content meet the interests of workers.

### 5.3. Strengths and Limitations

This study offers unique perspectives on the importance of work value perception and extends the factors affecting workers’ health. Indeed, the influence of work value perception has not been extensively explored in the literature. China has been described as “the world’s factory”; thus, testing the effects of work value perception on the health of Chinese workers is especially valuable. This study’s specific contributions are summarized as follows.

First, in the many studies that focus on improving the situation of workers, we observe the significance of workers’ ideologies and their perception of work value. With the coming of the Enlightenment and the Industrial Revolution in the seventeenth and eighteenth centuries, people began to focus on ideological emancipation and liberty [104,105]. Today, however, with rapid economic development, workers face intense competition as well as the possibility of being substituted by AI in more and more industries. It is against this background that this study aimed to open up a new research direction in the investigation of workers’ ideologies, especially in the context of emerging economies. After all, workers in factories are not machines but humans with ideas and emotions.

Second, we built our research model based on individual needs. Specifically, we combined ERG theory with work value perception to examine the mechanism of individual needs in relation to workers’ work value perception. We have emphasized that the pursuit of value is the driving force of an individual’s work and life, and the purpose of such pursuits is to meet one’s inner needs. In this way, this study not only enriches the application scope of the theory of individual needs but also provides a basis for studying work value perception and its consequences.

Third, with regard to methodology, previous studies have tended to overlook the problem of endogeneity between workers’ previous health status and the factors affecting their health outcomes. This study used IV regression to determine the net effect of work value perception on workers’ health. Moreover, since we used a large dataset of 16,890 respondents representing different professions, sectors, and regions, our results can be considered robust across all of China. The results showed that the effects of work value perception on workers’ physical and mental health were the same for different groups (e.g., different genders, marital statuses, and religious beliefs). In short, work value perception had significant positive effects on the health of all groups.

This study has some limitations. First, this was a cross-sectional study. Thus, the results do not reflect dynamic changes in the effects of work value perception on workers’ health. In the future, more dynamic results can be obtained using panel data. Second, the measurement of health could have been biased because it relied on self-rated physical and mental health. In future research, more objective measurements of health (e.g., medical records and records of mental illness) could be used to obtain individuals’ health status. Third, the work value perception scale is a newly developed scale, and its effectiveness needs further verification. Third, although we tested the impacts of work value perception on the physical and mental health of different subgroups, including gender, marital status, and religion belief groups, the obvious heterogeneity was not revealed. Future research could widen the subgroups, such as utilizing different working post groups, to examine the heterogeneous impact of work value perception on workers’ physical and mental health.

## 6. Conclusions

This study provides a theorical analysis framework related to work value perception and workers’ health based on ERG theory. Using big data, we empirically identify how work value perception has marked effects on workers’ physical and mental health through the mediating effect of life satisfaction. These findings add new evidence, from the Chinese context, regarding work value perception to the theory of labor health and welfare in labor economics research. This study may help to expand research on worker health from the material level to the spiritual level, offering implications of both domestic and international relevance.

## Figures and Tables

**Table 1:** Descriptive analysis.

Variable	Mean	SD	Min	Max
Physical health	3.589	0.994	1	5
Mental health	27.121	8.899	20	80
Work value perception	21.859	4.126	6	30
Life satisfaction	3.714	0.935	1	5
Gender (1 = male; 0 = female)	0.502	0.500	0	1
Age (years)	46.858	12.898	16	96
Education (years)	8.658	4.200	0	23
Marital status (1 = married; 0 = unmarried)	0.874	0.331	0	1
Religion (1 = with religion; 0 = without religion)	0.127	0.333	0	1
Income (logarithm of annual income)	9.910	1.141	4.605	14.931
Smoking (1 = yes; 0 = no)	0.308	0.462	0	1
Drinking (1 = yes; 0 = no)	0.222	0.415	0	1
Exercise (1 = yes; 0 = no)	0.314	0.464	0	1
IV	21.859	0.660	20.208	23.089
*N*	16,890			

**Table 2:** Benchmark regression.

Variables	Physical Health (Ordered Probit)			Mental Health (OLS)		
	(1)	(2)	(3)	(4)	(5)	(6)
Work value perception	0.028 ***	0.028 ***	0.012 ***	−0.241 ***	−0.239 ***	−0.091 ***
	(0.002)	(0.002)	(0.002)	(0.017)	(0.017)	(0.016)
Gender			0.103 ***			−1.471 ***
			(0.022)			(0.173)
Age			−0.027 ***			0.024 ***
			(0.001)			(0.006)
Education			0.019 ***			−0.156 ***
			(0.003)			(0.019)
Marital status			0.065 **			−1.019 ***
			(0.026)			(0.205)
Religion			−0.040			1.032 ***
			(0.027)			(0.212)
Income			0.049 ***			−0.214 ***
			(0.008)			(0.066)
Smoking			0.037			0.077
			(0.023)			(0.183)
Drinking			0.065 ***			0.039
			(0.023)			(0.177)
Exercise			0.036 *			−0.193
			(0.019)			(0.149)
Life satisfaction			0.266 ***			−2.466 ***
			(0.009)			(0.073)
Region		Yes	Yes		Yes	Yes
Pseudo R^2^	0.004	0.019	0.082			
R-squared				0.013	0.029	0.115
*N*	16,890					

Note: standard errors in parentheses; *** *p* < 0.01, ** *p* < 0.05, * *p* < 0.1; “Yes” means the variable is added to the model.

**Table 3:** Effects of work value perception on workers’ health (IV-ordered probit and IV−2 SLS models).

Variables	IV−2 SLS	IV-Ordered Probit	IV−2 SLS
	Physical Health	Physical Health	Mental Health
Work value perception	0.012 ***	0.015 ***	−0.095 ***
	(0.002)	(0.003)	(0.022)
Gender	0.083 ***	0.103 ***	−1.471 ***
	(0.018)	(0.022)	(0.171)
Age	−0.022 ***	−0.027 ***	0.024 ***
	(0.001)	(0.001)	(0.006)
Education	0.017 ***	0.019 ***	−0.156 ***
	(0.002)	(0.003)	(0.020)
Marital status	0.065 ***	0.065 **	−1.019 ***
	(0.021)	(0.026)	(0.212)
Religion	−0.036	−0.040	1.033 ***
	(0.023)	(0.027)	(0.214)
Income	0.043 ***	0.049 ***	−0.213 ***
	(0.007)	(0.008)	(0.070)
Smoking	0.029	0.037	0.078
	(0.019)	(0.023)	(0.176)
Drinking	0.058 ***	0.065 ***	0.040
	(0.019)	(0.023)	(0.170)
Exercise	0.029 *	0.035*	−0.191
	(0.016)	(0.019)	(0.146)
Life satisfaction	0.218 ***	0.264 ***	−2.462 ***
	(0.008)	(0.010)	(0.088)
Region	Yes	Yes	Yes
atanhrho_12		−0.020 *	
		(0.010)	
R-squared	0.202		0.115
*N*	16,890		

Note: standard errors in parentheses; *** *p* < 0.01, ** *p* < 0.05, * *p* < 0.1; “Yes” means the variable is added to the model.

**Table 4:** Impacts of different work value perception dimensions on workers’ physical health (IV-ordered probit).

Variables	(1)	(2)	(3)	(4)	(5)	(6)
Economic value perception	0.285 ***					
	(0.047)					
Emotional value perception		0.091 ***				
		(0.015)				
Social value perception			0.079 ***			
			(0.013)			
Respect value perception				0.079 ***		
				(0.013)		
Ability value perception					0.078 ***	
					(0.013)	
Interest value perception						0.077 ***
						(0.013)
Gender	0.091 ***	0.107 ***	0.103 ***	0.106 ***	0.102 ***	0.101 ***
	(0.022)	(0.022)	(0.022)	(0.022)	(0.022)	(0.022)
Age	−0.027 ***	−0.027 ***	−0.026 ***	−0.026 ***	−0.027 ***	−0.027 ***
	(0.001)	(0.001)	(0.001)	(0.001)	(0.001)	(0.001)
Education	0.023 ***	0.020 ***	0.019 ***	0.018 ***	0.019 ***	0.019 ***
	(0.003)	(0.003)	(0.003)	(0.003)	(0.003)	(0.003)
Marital status	0.027	0.063 **	0.067 **	0.066 **	0.068 **	0.069 ***
	(0.027)	(0.026)	(0.026)	(0.026)	(0.026)	(0.026)
Religion	−0.027	−0.037	−0.041	−0.040	−0.042	−0.042
	(0.027)	(0.027)	(0.027)	(0.027)	(0.027)	(0.027)
Income	0.050 ***	0.051 ***	0.047 ***	0.048 ***	0.048 ***	0.048 ***
	(0.008)	(0.008)	(0.009)	(0.008)	(0.008)	(0.008)
Smoking	0.024	0.039	0.035	0.037	0.037	0.039*
	(0.024)	(0.023)	(0.023)	(0.023)	(0.023)	(0.023)
Drinking	0.078 ***	0.064 ***	0.067 ***	0.063 ***	0.064 ***	0.063 ***
	(0.023)	(0.023)	(0.023)	(0.023)	(0.023)	(0.023)
Exercise	0.063 ***	0.037*	0.031	0.033 *	0.034 *	0.033 *
	(0.019)	(0.019)	(0.019)	(0.019)	(0.019)	(0.019)
Life satisfaction	0.270 ***	0.264 ***	0.265 ***	0.263 ***	0.261 ***	0.260 ***
	(0.010)	(0.010)	(0.010)	(0.010)	(0.010)	(0.010)
Region	Yes	Yes	Yes	Yes	Yes	Yes
atanhrho_12	−0.237 ***	−0.066 ***	−0.047 ***	−0.047 ***	−0.034 ***	−0.035 ***
	(0.038)	(0.014)	(0.013)	(0.012)	(0.013)	(0.013)
*N*	16,890					

Note: standard errors in parentheses; *** *p* < 0.01, ** *p* < 0.05, * *p* < 0.1; “Yes” means the variable is added to the model.

**Table 5:** Impacts of different work value perception dimensions on workers’ mental health (IV−2 SLS).

Variables	(1)	(2)	(3)	(4)	(5)	(6)
Economic value perception	−1.824 ***					
	(0.422)					
Emotional value perception		−0.566 ***				
		(0.130)				
Social value perception			−0.489 ***			
			(0.112)			
Respect value perception				−0.492 ***		
				(0.113)		
Ability value perception					−0.488 ***	
					(0.112)	
Interest value perception						−0.482 ***
						(0.111)
Gender	−1.413 ***	−1.494 ***	−1.471 ***	−1.487 ***	−1.460 ***	−1.459 ***
	(0.174)	(0.171)	(0.171)	(0.171)	(0.171)	(0.171)
Age	0.032 ***	0.025 ***	0.021 ***	0.023 ***	0.025 ***	0.024 ***
	(0.006)	(0.006)	(0.006)	(0.006)	(0.006)	(0.006)
Education	−0.186 ***	−0.161 ***	−0.152 ***	−0.151 ***	−0.153 ***	−0.155 ***
	(0.021)	(0.020)	(0.020)	(0.021)	(0.020)	(0.020)
Marital status	−0.790 ***	−1.011 ***	−1.031 ***	−1.025 ***	−1.040 ***	−1.047 ***
	(0.221)	(0.212)	(0.212)	(0.212)	(0.212)	(0.212)
Religion	0.953 ***	1.015 ***	1.040 ***	1.033 ***	1.047 ***	1.048 ***
	(0.218)	(0.215)	(0.215)	(0.215)	(0.215)	(0.215)
Income	−0.231 ***	−0.230 ***	−0.203 ***	−0.210 ***	−0.210 ***	−0.208 ***
	(0.071)	(0.070)	(0.071)	(0.070)	(0.070)	(0.071)
Smoking	0.156	0.066	0.087	0.077	0.074	0.063
	(0.179)	(0.176)	(0.176)	(0.176)	(0.176)	(0.176)
Drinking	−0.054	0.046	0.029	0.050	0.046	0.053
	(0.173)	(0.171)	(0.171)	(0.171)	(0.170)	(0.171)
Exercise	−0.376 **	−0.203	−0.167	−0.178	−0.183	−0.175
	(0.152)	(0.146)	(0.147)	(0.146)	(0.146)	(0.146)
Life satisfaction	−2.551 ***	−2.468 ***	−2.471 ***	−2.461 ***	−2.449 ***	−2.444 ***
	(0.088)	(0.088)	(0.088)	(0.089)	(0.089)	(0.090)
Region	Yes	Yes	Yes	Yes	Yes	Yes
R-squared	0.093	0.112	0.113	0.114	0.114	0.114
*N*	16,890					

Note: standard errors in parentheses; *** *p* < 0.01, ** *p* < 0.05; “Yes” means the variable is added to the model.

**Table 6:** Impacts of work value perception on workers’ physical health among different subgroups (IV-ordered probit).

Variables	Gender Groups		Marital Status Groups		Religious Belief Groups	
	Male	Female	Married	Unmarried	Religious	Nonreligious
Work value perception	0.016 ***	0.014 ***	0.015 ***	0.019 **	0.028 ***	0.012 ***
	(0.004)	(0.004)	(0.003)	(0.008)	(0.008)	(0.003)
Age	−0.024 ***	−0.028 ***	0.089 ***	0.210 ***	0.122 **	0.097 ***
	(0.001)	(0.001)	(0.024)	(0.059)	(0.061)	(0.024)
Education	0.013 ***	0.023 ***	−0.027 ***	−0.027 ***	−0.024 ***	−0.027 ***
	(0.004)	(0.003)	(0.001)	(0.002)	(0.002)	(0.001)
Marital status	−0.043	0.159 ***	0.019 ***	0.029 ***	0.016 **	0.020 ***
	(0.037)	(0.039)	(0.003)	(0.007)	(0.007)	(0.003)
Religion	0.039	−0.097 ***	−0.027	−0.119	0.145*	0.052 *
	(0.042)	(0.036)	(0.029)	(0.076)	(0.075)	(0.028)
Income	0.082 ***	0.027 **	0.050 ***	0.039 *	0.029	0.049 ***
	(0.013)	(0.012)	(0.009)	(0.023)	(0.024)	(0.009)
Smoking	0.046 *	−0.076	0.035	0.061	0.163**	0.025
	(0.025)	(0.080)	(0.025)	(0.063)	(0.071)	(0.025)
Drinking	0.066 ***	0.059	0.060 **	0.111 *	0.007	0.074 ***
	(0.025)	(0.059)	(0.024)	(0.063)	(0.072)	(0.024)
Exercise	−0.002	0.076 ***	0.035 *	0.025	−0.005	0.043**
	(0.027)	(0.027)	(0.021)	(0.052)	(0.056)	(0.020)
Life satisfaction	0.263 ***	0.263 ***	0.260 ***	0.300 ***	0.281 ***	0.2635 ***
	(0.014)	(0.013)	(0.010)	(0.026)	(0.027)	(0.010)
Region	Yes	Yes	Yes	Yes	Yes	Yes
atanhrho_12	0.820 ***	−0.027 *	−0.019 *	−0.037	−0.113 ***	−0.006
	(0.008)	(0.014)	(0.011)	(0.030)	(0.030)	(0.011)
*N*	8473	8417	14,768	2122	2146	14,744

Note: standard errors in parentheses; *** *p* < 0.01, ** *p* < 0.05, * *p* < 0.1; “Yes” means the variable is added to the model.

**Table 7:** Impacts of work value perception on workers’ mental health among different subgroups (IV−2 SLS).

Variables	Gender Groups		Marital Status Groups		Religious Belief Groups	
	Male	Female	Married	Unmarried	Religious	Nonreligious
Work value perception	−0.118 ***	−0.073 **	−0.091 ***	−0.1220 *	−0.256 ***	−0.070 ***
	(0.029)	(0.032)	(0.023)	(0.063)	(0.065)	(0.023)
Age	0.015 *	0.030 ***	−1.504 ***	−1.565 ***	−2.139 ***	−1.374 ***
	(0.008)	(0.009)	(0.186)	(0.458)	(0.477)	(0.184)
Education	−0.107 ***	−0.181 ***	0.026 ***	0.006	0.024	0.023 ***
	(0.030)	(0.028)	(0.007)	(0.014)	(0.016)	(0.006)
Marital status	−0.506 *	−1.509 ***	−0.147 ***	−0.272 ***	−0.145 **	−0.160 ***
	(0.278)	(0.331)	(0.022)	(0.062)	(0.059)	(0.022)
Religion	0.315	1.618 ***	0.881 ***	2.226 ***	−2.243 ***	−0.831 ***
	(0.300)	(0.302)	(0.226)	(0.650)	(0.630)	(0.225)
Income	−0.337 ***	−0.142	−0.220 ***	−0.180	−0.039	−0.234 ***
	(0.093)	(0.104)	(0.077)	(0.178)	(0.190)	(0.076)
Smoking	0.133	1.030	0.252	−1.038 **	−0.820 *	0.174
	(0.180)	(0.704)	(0.191)	(0.452)	(0.490)	(0.187)
Drinking	−0.184	1.052 **	0.061	−0.069	0.593	−0.044
	(0.180)	(0.487)	(0.182)	(0.480)	(0.473)	(0.182)
Exercise	0.226	−0.642 ***	−0.240	0.073	−0.309	−0.176
	(0.201)	(0.213)	(0.158)	(0.385)	(0.414)	(0.156)
Life satisfaction	−1.983 ***	−2.941 ***	−2.399 ***	−2.852 ***	−2.639 ***	−2.446 ***
	(0.118)	(0.130)	(0.096)	(0.225)	(0.249)	(0.094)
Region	Yes	Yes	Yes	Yes	Yes	Yes
R-squared	0.084	0.138	0.110	0.166	0.161	0.110
*N*	8473	8417	14,768	2122	2146	14,744

Note: robust standard errors in parentheses; *** *p* < 0.01, ** *p* < 0.05, * *p* < 0.1; “Yes” means the variable is added to the model.

**Table 8:** Mediating effect of life satisfaction in impacts of work value perception on workers’ physical health.

Variables	(1)	(2)	(3)	(4)	(5)	(6)	(7)
	Total Sample	Male	Female	Married	Unmarried	Religious	Nonreligious
Indirect effect	0.011 ***	0.012 ***	0.010 ***	0.011 ***	0.0139 ***	0.010 ***	0.011 ***
	(0.001)	(0.001)	(0.001)	(0.001)	(0.002)	(0.002)	(0.001)
Direct effect	0.009 ***	0.010 ***	0.008 ***	0.009 ***	0.011 **	0.016 ***	0.008 ***
	(0.002)	(0.003)	(0.003)	(0.002)	(0.005)	(0.005)	(0.002)
*N*	16,890	8473	8417	14,768	2122	2146	14,744

Note: standard errors in parentheses; *** *p* < 0.01, ** *p* < 0.05.

**Table 9:** Mediating effect of life satisfaction in impacts of work value perception on workers’ mental health.

Variables	(1)	(2)	(3)	(4)	(5)	(6)	(7)
	Total Sample	Male	Female	Married	Unmarried	Religious	Nonreligious
Indirect effect	−0.111 ***	−0.093 ***	−0.126 ***	−0.105 ***	−0.161 ***	−0.109 ***	−0.111 ***
	(0.006)	(0.008)	(0.010)	(0.007)	(0.021)	(0.017)	(0.006)
Direct effect	−0.089 ***	−0.108 ***	−0.071 ***	−0.087 ***	−0.103 **	−0.198 ***	−0.073 ***
	(0.018)	(0.024)	(0.027)	(0.018)	(0.047)	(0.056)	(0.018)
*N*	16,890	8473	8417	14,768	2122	2146	14,744

Note: standard errors in parentheses; *** *p* < 0.01, ** *p* < 0.05.

## Data Availability

Restrictions apply to the availability of these data. Data were obtained from the Center for Social Science Survey at Sun Yat-sen University in Guangzhou, China and are available from cssdata@mail.sysu.edu.cn with the permission of the Center for Social Science Survey at Sun Yat-sen University in Guangzhou, China.

## Appendix A

**Table A1:** Polychoric Correlation Coefficient Test on Items of CES-D.

Items	Item 1	Item 2	Item 3	Item 4	Item 5	Item 6	Item 7	Item 8	Item 9	Item 10	Item 11	Item 12	Item 13	Item 14	Item 15	Item 16	Item 17	Item 18	Item 19	Item 20
Item 1	1.000																			
Item 2	0.562	1.000																		
Item 3	0.690	0.636	1.000																	
Item 4	0.582	0.521	0.700	1.000																
Item 5	0.588	0.548	0.672	0.678	1.000															
Item 6	0.685	0.582	0.724	0.695	0.751	1.000														
Item 7	0.580	0.540	0.652	0.668	0.701	0.749	1.000													
Item 8	0.564	0.499	0.651	0.710	0.663	0.720	0.723	1.000												
Item 9	0.568	0.499	0.664	0.732	0.664	0.719	0.708	0.854	1.000											
Item 10	0.586	0.549	0.685	0.656	0.669	0.700	0.662	0.722	0.765	1.000										
Item 11	0.466	0.530	0.503	0.466	0.506	0.553	0.519	0.493	0.512	0.561	1.000									
Item 12	0.663	0.550	0.681	0.641	0.649	0.780	0.680	0.686	0.702	0.701	0.619	1.000								
Item 13	0.563	0.534	0.653	0.624	0.643	0.696	0.652	0.672	0.690	0.688	0.542	0.756	1.000							
Item 14	0.576	0.527	0.665	0.648	0.643	0.705	0.651	0.694	0.725	0.738	0.550	0.739	0.760	1.000						
Item 15	0.569	0.517	0.670	0.655	0.651	0.687	0.665	0.704	0.729	0.731	0.515	0.709	0.735	0.789	1.000					
Item 16	0.575	0.515	0.676	0.685	0.668	0.711	0.672	0.762	0.780	0.741	0.531	0.734	0.727	0.790	0.813	1.000				
Item 17	0.565	0.516	0.651	0.598	0.618	0.663	0.586	0.637	0.658	0.742	0.508	0.679	0.675	0.716	0.729	0.746	1.000			
Item 18	0.628	0.537	0.674	0.663	0.645	0.761	0.677	0.694	0.712	0.707	0.558	0.771	0.701	0.735	0.718	0.765	0.750	1.000		
Item 19	0.569	0.516	0.663	0.665	0.668	0.681	0.658	0.710	0.733	0.745	0.509	0.700	0.720	0.765	0.842	0.796	0.773	0.763	1.000	
Item 20	0.583	0.546	0.687	0.680	0.671	0.698	0.674	0.758	0.782	0.765	0.540	0.711	0.726	0.774	0.801	0.847	0.791	0.777	0.851	1.000

## Appendix B

### Measurement Dimensions of Work Value Perception

To what extent do you agree with the following statements?

1. My current job can meet my economic needs. (Economic value perception)
2. My current job can make me feel at ease. (Emotional value perception)
3. My current job can make me get to know more people. (Social value perception)
4. My current job can make me get respect from other people. (Respect value perception)
5. My current job can give my ability to full play. (Ability value perception)
6. My current job can satisfy my interest. (Interest value perception)
